# The characteristic of asthma control among nasal diseases population: Results from a cross-sectional study

**DOI:** 10.1371/journal.pone.0191543

**Published:** 2018-02-22

**Authors:** Jiangtao Lin, Jie Gao, Kefang Lai, Xin Zhou, Bei He, Jianying Zhou, Changzheng Wang

**Affiliations:** 1 China-Japan Friendship Hospital, Beijing, China; 2 Department of Outcomes Research, MSD China, Shanghai, China; 3 The First Affiliated Hospital of Guangzhou Medical University, Guangzhou, China; 4 Shanghai General Hospital, Shanghai, China; 5 Peking University Third Hospital, Beijing, China; 6 The First Affiliated Hospital, Zhejiang University, Zhejiang, China; 7 The Second Affiliated Hospital, The Third Military Medical University, Chongqing, China; Forschungszentrum Borstel Leibniz-Zentrum fur Medizin und Biowissenschaften, GERMANY

## Abstract

Asthma affects a large number of patients in China, but comprehensive evaluation of risks associated with poor asthma control in asthmatic patients with nasal diseases was still limited. We conducted this study to provide a comprehensive estimate of asthma control in Chinese asthma patients with combined nasal diseases, to explore the effect of kinds of nasal diseases on the asthma control, and to identify risk factors associated with uncontrolled asthmatic patients with combined nasal diseases. 1756 asthma patients concomitant with nasal diseases aged ≥17 years and representing all regions of mainland China were surveyed. Multivariate logistic regression model with all related demographic characteristics and disease characteristics factors entered was used to identify risk factors associated with uncontrolled asthma. 56.4% of asthmatic patients with combined allergic rhinitis or rhinosinusitis or rhinopolyp remained uncontrolled. Concomitant without allergic rhinitis, younger age, better treatment adherence and higher education level might positively impact asthma control among asthmatic patients with combined nasal diseases. Perennial allergic rhinitis (OR = 1.5, P = 0.021), moderate-severe allergic rhinitis (OR = 2.2, P = 0.001) were all found to significantly increase the risk of uncontrolled asthma among asthma patients with combined allergic rhinitis. The high prevalence of uncontrolled asthma indicates that asthma management among adult Chinese asthmatic patients comorbid with nasal disease is still a challenge. Efforts should be made to raise the awareness of asthma management and to provide sufficient treatment will greatly contribute to improved quality of asthma management. It is possible to minimize the influence of allergic rhinitis on asthma control by improving nasal function, especially for more severe allergic rhinitis and perennial allergic rhinitis.

## Introduction

Asthma and nasal diseases (consisting of rhinitis, rhinosinusitis, and rhinopolyp) [1], are highly prevalent chronic respiratory diseases. Studies indicate that these diseases impact on patients’ social life, sleep quality, school performance, and work productivity [2] and incur substantial economic costs [3]. According to World Health Organization (WHO), the prevalence of clinical asthma in China was at lower level worldwide (under 2.5%) while its case fatality rate was classified as highest level (over 10 deaths per 100 000 people with asthma)[3] indicating a big challenge in asthma control in China. Traditionally asthma and nasal diseases have been considered as two different categories, affecting the lower and upper respiratory tract respectively. However, according to recent pathophysiological findings, they have been identified both disorders as manifestations of a single chronic inflammatory respiratory syndrome. They are linked by the united airways disease that encompasses commonalities in pathophysiology, epidemiology, and treatment [2, 4–7].

Epidemiologic studies have consistently reported that asthma and nasal diseases often coexist. Many studies indicate the prevalence of comorbid allergic rhinitis (AR) is relatively high and ranged from 24% to 94% among adults with asthma in Europe and USA [8–10]. Furthermore, a recommendation by the Allergic Rhinitis and its Impact on Asthma (ARIA) workshop in collaboration with the World Health Organization (WHO) -that combined treatment strategy can be used to treat upper and lower airway diseases [2]-indicates that appropriate combined treatment can lead to both improved AR and asthma. Besides, association between asthma and rhinosinusitis or rhinopolyp is also clearly established, but the exact mechanism is still controversial [11–12]. Approximately 20–33% of patients with chronic rhinosinusitis also manifest asthma, a prevalence of approximately four times greater than that of the general population [12–14]. Rhinosinusitis and asthma coexist and impact each other and proper medical and surgical management of rhinosinusitis in the asthmatic patient can result in both improved rhinosinusitis and asthmatic symptoms [15]. 71% of patients with rhinopolyp also had asthma [16]. And the presence of rhinopolyp was related to severity of asthma [17]. Evidences show that asthma and nasal diseases impact on one another at many different levels, and allergic rhinitis and sinonasal inflammation might be considered a risk factor for the worsening of asthma, while the exact nature of this relationship remains debated [5,9,15].

However, comprehensive evaluation of risks associated with poor asthma control in Chinese asthmatic patients concomitant with nasal diseases was still very limited. A study showed that 69.9% of Chinese asthma patients over the age of 14 years reported AR symptoms and that the presence and severity of AR were negatively associated with asthma control [18]. Due to the limited information on the state of asthma management in Chinese patients with comorbid nasal diseases and the high prevalence of comorbid nasal diseases, it is important to advise clinicians and patients about the best evidence based management of asthma in patients with concomitant nasal diseases in China. This study aims to provide a comprehensive estimate of asthma control in Chinese asthma patients with combined nasal diseases, to explore the effect of kinds of nasal diseases to the asthma control, and to identify risk factors associated with uncontrolled asthmatic patients with combined nasal diseases.

## Materials and methods

### Study design and patients

The study protocol was reviewed and approved by investigational center ethics committee at China-Japan Friendship Hospital, Beijing, China. The study was designed, conducted and reported in accordance with International Conference on Harmonization of Technical Requirements for Registration of Pharmaceuticals for Human Use (ICH) Harmonized Tripartite Guidelines for Good Clinical Practice, all applicable subject privacy requirements and the guiding principles of the 2013 Declaration of Helsinki.

This nationwide cross-sectional observational study was conducted in China from November, 2012 to June, 2013. A total of 8873 asthma patients aged ≥2 years who visited outpatient clinics of respiratory disease for treatment, prescription refill and consultation were enrolled during this study period. Data of 1756 asthma patients aged ≥17 with concomitant AR or rhinosinusitis or rhinopolyp among them will be used in this analysis.

The asthma patients aged ≥17 were consecutively enrolled from 48 tertiary general hospitals in 34 cities of different provinces across China, covering all territories and regions of mainland China except Tibet. All participants had confirmed asthma for 3 months at least and had symptoms or took asthma medication during the past 12 months. Asthma diagnosis was confirmed by medical chart review at the enrollment visit. According to the Global Initiative for Asthma (GINA) [19–21], definition of asthma is a heterogeneous disease, usually characterized by chronic airway inflammation. It is defined by the history of respiratory symptoms such as wheeze, shortness of breath, chest tightness and cough that vary over time and in intensity, together with pulmonary function tests demonstrating variable airflow limitation by means of airway responsiveness testing or airway reversibility testing.

Asthma duration and treatment were also confirmed by medical chart review. Eligible patients were requested to sign the informed consent. Patients who had intermittent asthma, COPD, bronchiectasis, bronchitis, cystic fibrosis, lung cancer or pneumonia or who were not able to fill in the questionnaires were excluded. Allergic rhinitis was defined by the presence of typical AR symptoms (i.e., watery rhinorrhea, sneezing, nasal obstruction and nasal pruritis), based on the ARIA criteria [2], accompanied by eye-associated symptoms (tearing, burning or itching). Rhinosinusitis or rhinopolyp diagnosis was confirmed by medical chart review and it was defined by an accurate and thorough history and physical examination including nasal endoscopy and nasosinus computed tomography (CT) scanning. In this study, if the terminology AR, rhinosinusitis, rhinopolyp appeared alone, then it indicated that patients only had AR, rhinosinusitis, or rhinopolyp disease. If the asthma patients had ≥2 nasal diseases, we would use such expression like ‘Rhinopolyp + Rhinosinusitis’ to indicate the patients had rhinosinusitis and rhinopolyp disease.

The study was originally approved by the Independent Ethics Committee of China-Japan Friendship Hospital. Other participating hospitals either accepted the decision of that committee or conducted a further independent ethics review, according to their own institutional policy.

### Data collection

Socio-demographic and clinical information, including family history of allergic disease, concomitant disease (e.g., allergic rhinitis, rhinosinusitis, rhinopolyp), asthma symptoms, asthma duration since diagnosis, asthma related treatments and tests and asthma treatment adherence, was collected from participants’ medical charts during their visits. Any identifier to individual patient, such as social security number, identification number and full name, was not collected. Body Mass Index (BMI, kg/m^2^) was measured and classified as normal (18.5≤BMI<24), lean (BMI<18.5), overweight (24≤BMI<28), and obesity (BMI≥28) based on Criteria of Weight for Adults issued by National Health and Family Planning Commission of the People’s Republic of China in 2013 [22]. Asthma treatment adherence was assessed by physicians through review of controller prescription refill in the past 3 months and was classified into 4 levels: complete adherence (≥90%), good adherence (70–89%), poor adherence (50–69%) and non-adherence (<50%).

The five-item Asthma Control Test (ACT) questionnaire was used to assess the level of asthma control [23–24]. Asthma control was classified into controlled (ACT≥20) and uncontrolled (ACT≤19) [25]. The questionnaire was completed by participants during the interview and reviewed by physicians for completeness.

### Quality control

All data were collected using a uniform data collection form. All investigators were trained on the standardized study protocol and data collection form before initiation of the study. A Clinical Research Organization (CRO) was employed to monitor the quality of data collection and to manage the data. All data were input into a programmed database by two persons, independently, for statistical analysis.

### Statistical analysis

Descriptive statistical analysis of uncontrolled asthmatic patients’ number, rate and 95% CI were presented by demographic characteristics of different subgroups with nasal disease. To evaluate the association between clinical or disease categories, statistical analyses were performed using Chi-Square test to compare qualitative variables between 2 categories for uncontrolled asthma. Cochran-Armitage test was used to detect the trend of proportions of uncontrolled asthma in patients among more than 2 ordinal categories. A two times multivariate logistic regression model was used to identify the risk factors associated with uncontrolled asthma and to derive odds ratio (OR) and the 95% confidence interval of each factor. All related demographic characteristics and disease characteristics factors entered the multivariate logistic regression models. A two-tailed P-value of less than 0.05 was considered to be statistically significant.

Statistical analyses were performed using the SAS® System for Windows TM 9.2 (SAS Institute Inc., Cary, NC, USA.).

## Results

### Demographic characteristics of uncontrolled asthmatic patients with combined nasal diseases

Overall, more than half (56.4%, 990/1756) of asthmatic patients with combined AR or rhinosinusitis or rhinopolyp remained uncontrolled (ACT≤19). To explore the differences among patients with different nasal diseases, the data was analyzed by comparison through four mutual exclusive groups: combined with AR, rhinosinusitis, rhinopolyp and combined with ≥2 out of 3 foregoing nasal diseases. The frequency of uncontrolled asthma for these four groups were 57.1% (691/1211), 45.2% (61/135), 56.5% (26/46) and 58.2% (212/364), respectively.

Significant statistical difference was found in common among age groups, education levels, asthma treatment and asthma treatment adherence levels according to demographic factors analysis of asthmatic patients with nasal diseases (see Table 1).

**Table 1:** Distribution of demographics, uncontrolled asthma for asthmatic patients with nasal diseases (N = 1756).

Factor	Combined with AR (N = 1211)		Combined with Rhinosinusitis (N = 135)		Combined with Rhinopolyp (N = 46)		Combined with ≥2 nasal diseases (N = 364)	
	n^a^/Nx^b^	Rate (95% CI) /P-value^c^	n^a^/Nx^b^	Rate (95% CI)/P-value^c^	n^a^/Nx^b^	Rate (95% CI)/P-value^c^	n^a^/Nx^b^	Rate (95% CI)/P-value^c^
**Overall**	691/1211	57.06(54.27, 59.85)	61/135	45.19(36.79, 53.58)	26/46	56.52 (42.20, 70.85)	212/364	58.24(53.18, 63.31)
**Age group (years)**		<0.001^d^		0.077		0.032^d^		0.148
17–29	89/199	44.72 (37.82, 51.63)	5/20	25.00 (6.02, 43.98)	0/3	0 (NA,NA)	27/48	56.25 (42.22, 70.28)
30–44	222/408	54.41 (49.58, 59.24)	21/48	43.75 (29.72, 57.78)	8/17	47.06 (23.33, 70.79)	67/111	60.36 (51.26, 69.46)
45–59	275/432	63.66 (59.12, 68.19)	22/43	51.16 (36.22, 66.10)	13/20	65.00 (44.10, 85.90)	93/146	63.70 (55.90, 71.50)
60–70	78/132	59.09 (50.70, 67.48)	10/17	58.82 (35.43, 82.22)	5/5	100 (NA,NA)	21/46	45.65 (31.26, 60.05)
>70	27/40	67.50 (52.99, 82.01)	3/7	42.86 (6.20, 79.52)	0/1	0 (NA,NA)	4/13	30.77 (5.68, 55.86)
**Gender**		0.073		0.486		0.003^d^		0.914
Male	249/463	53.78 (49.24, 58.32)	29/59	49.15 (36.40, 61.91)	9/25	36.00 (17.18, 54.82)	85/147	57.82 (49.84, 65.81)
Female	442/748	59.09 (55.57, 62.61)	32/76	42.11 (31.01, 53.21)	17/21	80.95 (64.16, 97.75)	127/217	58.53 (51.97, 65.08)
**BMI (kg/m^2)^e^**		0.065		0.185		0.991		0.355
18.5≤ BMI<24 (normal)	357/652	54.75 (50.93, 58.58)	39/77	50.65 (39.48, 61.82)	12/22	54.55 (33.74, 75.35)	105/185	56.76 (49.62, 63.90)
BMI<18.5 (lean)	38/66	57.58 (45.65, 69.50)	2/5	40.00 (0.00, 82.94)	2/2	100 (NA,NA)	11/16	68.75 (46.04, 91.46)
24≤BMI<28 (overweight)	235/386	60.88 (56.01, 65.75)	16/44	36.36 (22.15, 50.58)	8/15	53.33 (28.09, 78.58)	66/120	55.00 (46.10, 63.90)
BMI≥28 (obese)	60/101	59.41 (49.83, 68.98)	4/9	44.44 (11.98, 76.91)	4/7	57.14 (20.48, 93.80)	30/42	71.43 (57.77, 85.09)
**Smoking status**		0.217		0.141		0.001^d^		0.79
Never smoked	549/975	56.31 (53.19, 59.42)	46/110	41.82 (32.60, 51.04)	23/32	71.88 (56.30, 87.45)	173/295	58.64 (53.02, 64.26)
Ex-smoker	94/160	58.75 (51.12, 66.38)	11/18	61.11 (38.59, 83.63)	3/8	37.50 (3.95, 71.05)	26/51	50.98 (37.26, 64.70)
Current smoker	48/76	63.16 (52.31, 74.00)	4/7	57.14 (20.48, 93.80)	0/6	0 (NA,NA)	13/18	72.22 (51.53, 92.91)
**Education**		<0.001^d^		0.010^d^		0.162		0.236
Primary school and below	99/147	67.35 (59.77, 74.93)	7/10	70.00 (41.60, 98.40)	7/9	77.78 (50.62, 100)	24/40	60.00 (44.82, 75.18)
Junior high school	163/271	60.15 (54.32, 65.98)	14/29	48.28 (30.09, 66.46)	4/9	44.44 (11.98, 76.91)	51/85	60.00 (49.59, 70.41)
Senior high school	205/329	62.31 (57.07, 67.55)	24/42	57.14 (42.18, 72.11)	10/15	66.67 (42.81, 90.52)	64/99	64.65 (55.23, 74.06)
College and above	224/464	48.28 (43.73, 52.82)	16/54	29.63 (17.45, 41.81)	5/13	38.46 (12.02, 64.91)	73/140	52.14 (43.87, 60.42)
**First-degree relative(s) with asthma**		0.294		0.087		0.509		0.11
No	531/944	56.25 (53.09, 59.41)	48/115	41.74 (32.73, 50.75)	18/34	52.94 (36.16, 69.72)	152/273	55.68 (49.78, 61.57)
Yes	160/267	59.93 (54.05, 65.80)	13/20	65.00 (44.10, 85.90)	8/12	66.67 (39.99, 93.34)	60/91	65.93 (56.20, 75.67)
**Asthma duration >3 years since diagnosis**^**e**^		0.383		0.294		0.766		0.397
No	356/638	55.80 (51.95, 59.65)	34/82	41.46 (30.80, 52.13)	11/21	52.38 (31.02, 73.74)	99/177	55.93 (48.62, 63.25)
Yes	334/572	58.39 (54.35, 62.43)	27/37	50.94 (37.48, 64.40)	15/25	60.00 (40.80, 79.20)	113/187	60.43 (53.03, 67.44)
**Asthma treatment** **(in past 4 weeks)**		0.003 ^d^		1.000		0.828		0.521
No treatment	142/214	66.36(59.60, 72.65)	6/11	54.55(23.38, 83.25)	15/30	50.00(31.30, 68.70)	33/51	64.71(50.07, 77.57)
ICS^e^ treatment	507/932	54.40(51.14, 57.63)	19/34	55.88(37.89, 72.81)	44/101	43.56(33.72, 53.80)	171/301	56.81(51.00, 62.48)
Other treatment	42/65	64.62(51.77, 76.08)	1/1	100.00(2.50, 100.00)	2/4	50.00(6.76, 93.24)	8/12	66.67(34.89, 90.08)
**Asthma treatment adherence**^**f**^		<0.001^d^		0.011^d^		0.891		<0.001
Complete adherence	197/475	41.47 (37.04, 45.90)	18/57	31.58 (19.51, 43.65)	9/16	56.25 (31.94, 80.56)	76/159	47.80 (40.03, 55.56)
Good adherence	178/315	56.51 (51.03, 61.98)	22/37	59.46 (43.64, 75.28)	10/17	58.82 (35.43, 82.22)	56/99	56.57 (46.80, 66.33)
Poor adherence	155/219	70.78 (64.75, 76.80)	13/32	40.63 (23.61, 57.64)	4/9	44.44 (11.98, 76.91)	46/64	71.88 (60.86, 82.89)
Non-adherence	160/201	79.60 (74.03, 85.17)	8/9	88.89 (68.36, 100.00)	3/4	75.00 (32.57, 100.00)	34/42	80.95 (69.08, 92.83)

Abbreviations: AR, allergic rhinitis; BMI, body mass index; CI, confidence interval; NA, not available.

^a^ number of uncontrolled asthma patients in each category.

^b^ number of non-missing data in each category.

^c^ For factors of two categories, p-value was calculated using chi-square test comparing proportions of uncontrolled asthma between the two categories.

For factors of more than two categories, p-value was calculated using Cochran-Armitage test to test the trend of proportions of uncontrolled asthma across

the ordinal categories.

^d^ means p < 0.05.

^**e**^ ICS: Inhaled Corticosteroids administered alone or combined with other medications.

^f^ indicates that subjects with missing value of that category.

Patients aged ≥45 years had more proportions in uncontrolled asthma than younger age groups for asthmatic patients combined with AR (63.7% in age 45–59, 59.1% in age 60–70 and 67.5% in age >70 vs. 44.7% in age 17–29 and 54.41% in age 30–44) or rhinopolyp (65.0% in age 45–59 and 100.0% in age 60–70 vs. 47.1% in age 30–44), which indicated that age <45 years was significantly associated with better asthma control, relative to elder age groups (p<0.001). However, because of the sparse data of rhinopolyp group this result might be unreliable.

Compared to educational level of primary school and below, the group with education level of college and above had the lower proportion of uncontrolled asthma for asthmatic patients with AR (48.3% vs. 67.4%, p<0.001) or rhinosinusitis (29.6% vs. 70.0%, p = 0.101). It showed that higher education level (i.e., college and above) was significantly conducive to control asthma better.

The ICS treatment achieved the lowest uncontrolled rate in the asthmatic patients combined with AR, rhinopolyp, or combined with ≥2 nasal disease with rate of 54.40%, 43.56%, 56.81%. The difference was statistically significant in asthmatic patients combined with AR.

There was a significantly common trend in asthmatic patients combined with AR, rhinosinusitis, or combined with ≥2 nasal diseases that the more complete asthma treatment adherence is, the less frequently the uncontrolled asthma occurs. The lowest rates of uncontrolled asthma were observed in complete adherence groups for these three categories of patients respectively, compared to non-adherence group (AR: 41.5% vs. 79.6%, p<0.001; rhinosinusitis: 31.6% vs. 88.9%, p = 0.011; with ≥2 nasal diseases: 47.8% vs. 81.0%, p<0.001). This trend was also found in patients with rhinopolyp alone although it was not statistically significant.

With regard to asthma patients combined with rhinopolyp, significant statistical difference was found between gender groups. Female patients were significantly worse in uncontrolled asthma, compared to male patients (81.0% vs. 36.0%, p = 0.003). It was also found that smoking status had significant effect on uncontrolled rate of asthma in this subgroup. Uncontrolled rates were calculated as 71.9%, 37.5% and 0.0% for nonsmoker, ex-smoker and current smoker respectively (p = 0.001), but this result might be irrational because of a paucity of data in smoker.

### Risk factors of uncontrolled asthma

From Table 1, we found that the rates of uncontrolled asthma had slight differences among patients with different nasal diseases. To explore these differences in further and to clarify which nasal disease may have significant impact on asthma control, we examined the uncontrolled rates in patients with ≥2 nasal diseases and all population in this study.

For asthmatic patients with 2 or 3 nasal diseases, it was disclosed that uncontrolled asthma was prone to be more in those whose nasal comorbidities including AR, although this finding was not statistically significant (AR + rhinopolyp 56.3%, AR + rhinosinusitis 60.5% and AR + rhinopolyp + rhinosinusitis 57.3% vs. rhinopolyp + rhinosinusitis 52.3%, p = 0.522). To determine whether this finding was accurate and whether specific nasal disease had potential impact on asthma control, we checked the rate of uncontrolled asthma through specifying each concomitant nasal disease in asthma patients with two nasal diseases (i.e., between patients with and without AR, with and without rhinosinusitis, and with and without rhinopolyp). It was found that there was little difference in uncontrolled rates between patients with and without rhinosinusitis (59.0% vs. 56.3%, p = 0.750), and greater difference of uncontrolled rate between patients with AR and without AR (59.7% vs. 52.3%, p = 0.406) or between patients with and without rhinopolyp (54.4% vs. 60.5%, p = 0.367) although the differences were not statistically significant (see Table 2). We deducted that different comorbid nasal diseases might have impact on asthma control to some extent and we decided to test it for all analysis population in multivariate logistic regression model.

**Table 2:** Uncontrolled rate distribution for asthmatic patients with ≥2 nasal diseases.

Population	n^a^/Nx^b^	Rate (95% CI)	P-value^c^
**Asthma patients with ≥2 nasal diseases (N = 364)**			
AR + Rhinopolyp (N = 48)	27/48	56.25 (42.22, 70.28)	0.522
AR + Rhinosinusitis (N = 190)	115/190	60.53 (53.58, 67.48)	
Rhinopolyp + Rhinosinusitis (N = 44)	23/44	52.27 (37.51, 67.03)	
AR + Rhinopolyp + Rhinosinusitis (N = 82)	47/82	57.32 (46.61, 68.02)	
**Asthma patients with 2 nasal diseases (N = 282)**			
Concomitant with AR (N = 238)	142/238	59.66 (53.43, 65.90)	0.406
Concomitant without AR (N = 44)	23/44	52.27 (37.51, 67.03)	
Concomitant with Rhinosinusitis (N = 234)	138/234	58.97 (52.67, 65.28)	0.750
Concomitant without Rhinosinusitis (N = 48)	27/48	56.25 (42.22, 70.28)	
Concomitant with Rhinopolyp (N = 92)	50/92	54.35 (44.17, 64.53)	0.367
Concomitant without Rhinopolyp (N = 190)	115/190	60.53 (53.58, 67.48)	

Abbreviations: AR, allergic rhinitis; CI, confidence interval.

^a^ number of uncontrolled asthma patients in each category.

^b^ number of non-missing data in each category.

^c^ For factors of two categories, p-value was calculated using chi-square test comparing proportions of uncontrolled asthma between the two categories. For factors of more than two categories, p-value was calculated using Cochran-Armitage test to test the trend of proportions of uncontrolled asthma across the ordinal categories.

Risk factor of uncontrolled rate among asthma patients with concomitant nasal diseases is presented in Table 3. All related common demographic factors found in Table 1 and disease characteristics factors entered the multivariate logistic regression model and it was found that the risk of uncontrolled asthma increased significantly when asthma treatment adherence decreased (p<0.001). Compared with complete adherence, the odds ratio (OR) of uncontrolled asthma was 1.7 [(1.3–2.2), p<0.001] among patients with good adherence, 2.9 [(2.2–3.8), p<0.001] and 5.2 [(3.6–7.3), p<0.001] for those with poor adherence and non-adherence, respectively. Additionally, age ≥45 years [OR = 1.3 (1.1–1.6), p = 0.016] was positively related with uncontrolled asthma. Patients with first-degree family history of asthma had 1.3 times more likely to be uncontrolled asthma than those without such family history [OR = 1.3 (1.0–1.7), p = 0.024]. However, education level of college and above, in contrast with primary school and below, was negatively related to uncontrolled asthma [OR = 0.6 (0.4–0.8), p = 0.002]. The indication that younger age, better treatment adherence and higher education level will positively impact asthma control is consistent with the findings in Table 1. Regarding the relationship between nasal disease and asthma control, it was disclosed that AR was a significant risk factor [OR = 1.5 (1.0–2.1), p = 0.029] for uncontrolled asthma but such finding was not obtained for the other nasal disease such as rhinosinusitis and rhinopolyp in this study.

**Table 3:** Risk factors of uncontrolled asthma among patients with concomitant AR or/with rhinosinusitis or/with rhinopolyp (N = 1756).

Factor	Odds Ratio (95% CI)^a^	p-value^a^
**Age ≥45 years**	1.30 (1.05, 1.61)	0.016^b^
**Female (vs. ‘male’)**	1.19 (0.92, 1.53)	0.183
**BMI (vs. ‘normal’)**		
<18.5 (lean)	1.11 (0.69, 1.77)	0.673
24–27.9 (overweight)	1.01 (0.81, 1.27)	0.903
≥28 (obese)	1.11 (0.77, 1.61)	0.565
**Current or previous smoking**	1.11 (0.82, 1.52)	0.493
**Education (vs. ‘Primary school and below’)**		
Junior high school	0.85 (0.58, 1.23)	0.387
Senior high school	0.99 (0.69, 1.43)	0.965
College and above	0.58 (0.40, 0.82)	0.002^b^
**First-degree relative(s) with asthma**	1.33 (1.04, 1.69)	0.024^b^
**Asthma duration >3 years since diagnosis**	1.19 (0.97, 1.47)	0.093
**Asthma treatment adherence (vs. ‘complete adherence’)**		
Good adherence	1.69 (1.33, 2.15)	<0.0001^b^
Poor adherence	2.87 (2.16, 3.81)	<0.0001^b^
Non-adherence	5.16 (3.64, 7.32)	<0.0001^b^
**Concomitant AR(vs. ‘no’)**	1.47 (1.04, 2.07)	0.029^b^
**Concomitant Rhinosinusitis(vs. ‘no’)**	1.14 (0.87, 1.48)	0.345
**Concomitant Rhinopolyp(vs. ‘no’)**	1.04 (0.75, 1.43)	0.820

Abbreviations: AR, allergic rhinitis; BMI, body mass index; CI, confidence interval; vs, versus.

^a^ odds-ratio and p-value were from a multivariate logistic regression model with uncontrolled asthma as the event and all factors listed in this table as the predictors.

^b^ means p < 0.05.

### Risk factors of uncontrolled asthma in combined AR patients

To find out more details on the influence of AR on asthma control, distribution of uncontrolled asthma and clinical characteristics in asthmatic patients with AR was analyzed (See Table 4). It was concluded that perennial AR and moderate-severe AR significantly led to worsen asthma control, respectively (60.5% vs. 54.0%, P = 0.023 and 60.0% vs. 45.8%, p<0.001). The proportion of uncontrolled asthma was significantly lower in those with AR treatment than without it (50.7% vs. 60.8%, p = 0.033).

**Table 4:** Distribution of uncontrolled asthma by AR, clinical characteristics among asthmatic patients with AR (N = 1211).

Population (N = 1211)	n^a^/Nx^b^	Rate (95% CI)	p-value^c^
**Overall**	691/1211	57.06 (54.27, 59.85)	
**Perennial vs. Seasonal AR**			0.023^d^
Perennial AR	342/565	60.53 (56.50, 64.56)	
Seasonal AR	349/646	54.02 (50.18, 57.87)	
**Duration of AR**			0.096
Persistent	189/309	61.17 (55.73, 66.60)	
Intermittent	502/902	55.65 (52.41, 58.90)	
**Severity of AR**			<0.001^d^
mild	113/247	45.75 (39.54, 51.96)	
moderate—severe	578/964	59.96 (56.87, 63.05)	
**Current AR treatment**			0.033^d^
Yes	286/564	50.71 (46.58, 54.84)	
No	90/148	60.81 (52.95, 68.68)	
Missing	315/499		
**Performed allergen skin prick test**			0.234
No	589/1019	57.80 (54.77, 60.83)	
Yes	102/192	53.13 (46.07, 60.18)	

Abbreviations: AR, allergic rhinitis; CI, confidence interval; vs, versus.

^a^ number of uncontrolled asthma patients in each category.

^b^ number of non-missing data in each category.

^c^ P-value was calculated using chi-square test comparing proportions of uncontrolled asthma between the two categories.

^d^ means p < 0.05.

Since AR was the one related to uncontrolled asthma compared to the other nasal diseases in this study, it was necessary to create another multivariate logistic regression model to investigate the AR-related predictors associated with asthma control in patients with asthma and AR (See Table 5). It was showed that perennial AR [OR = 1.5 (1.1–2.0), p = 0.021], moderate-severe AR [OR = 2.2 (1.4–3.5), p = 0.001] were all found to significantly increase the risk of uncontrolled asthma, supporting our conclusion in Table 4, other than age ≥45 years and asthma treatment adherence which we also proved relevant with asthma control in patients with nasal diseases.

**Table 5:** Risk factors of uncontrolled asthma among patients with concomitant AR (N = 1211).

Factor	Odds Ratio (95% CI)^a^	p-value^a^
**Age ≥45 years**	1.68 (1.19, 2.37)	0.003^b^
**Female (vs. ‘male’)**	1.28 (0.85, 1.94)	0.234
**BMI (vs. ‘normal’)**		
<18.5 (lean)	0.92 (0.43, 1.96)	0.821
24–27.9 (overweight)	1.14 (0.79, 1.63)	0.486
≥28 (obese)	0.82 (0.42, 1.58)	0.548
**Current or previous smoking**	1.35 (0.80, 2.27)	0.263
**Education (vs. ‘Primary school and below’)**		
Junior high school	1.16 (0.62, 2.21)	0.641
Senior high school	1.57 (0.86, 2.88)	0.144
College and above	0.86 (0.48, 1.54)	0.608
**First-degree relative(s) with asthma**	1.21 (0.82, 1.79)	0.328
**Asthma duration >3 years since diagnosis (vs. ‘No’)**	1.16 (0.84, 1.61)	0.361
**Asthma treatment adherence (vs. ‘complete adherence’)**		
Good adherence	1.33 (0.91, 1.95)	0.140
Poor adherence	2.83 (1.74, 4.58)	<0.0001^b^
Non-adherence	3.55 (2.06, 6.12)	<0.0001^b^
**Perennial AR (vs. seasonal AR)**	1.47 (1.06, 2.03)	0.021^b^
**Persistent AR (vs. ‘Intermittent AR’)**	1.17 (0.83, 1.66)	0.377
**Moderate-severe AR(vs. ‘Mild AR’)**	2.21 (1.39, 3.51)	0.001^b^
**Currently AR treatment(vs. ‘No’)**	0.71 (0.47, 1.06)	0.093
**Performed allergen skin prick test(vs. ‘No’)**	1.03 (0.71, 1.49)	0.894

Abbreviations: AR, allergic rhinitis; BMI, body mass index; CI, confidence interval; vs, versus.

^a^ odds-ratio and p-value were from a multivariate logistic regression model with uncontrolled asthma as the event and all factors listed in this table as the predictors.

^b^ means p < 0.05.

## Discussion

Epidemiologic studies have consistently showed that nasal diseases is common comorbidity of asthma. More than half (56.4%) of the adult Chinese asthma patients with combined nasal diseases surveyed in this study had uncontrolled asthma. According to an epidemiologic survey from February of 2010 to August of 2011, uncontrolled rate of asthma was 34.9% based on ACT questionnaire among 2034 asthma patients aged >14 from 164, 215 Chinese residents [26]. The prevalence of uncontrolled asthma among the general population was much lower than that rate in asthma patients with nasal diseases (34.9% vs. 56.4%), indicating that nasal disease may have significant negative impact on asthma control, so it is important to analyze the status of asthma control for the patients with both asthma and nasal diseases and provide insights for asthma disease management.

Several risk factors for uncontrolled asthma were identified in asthmatic patients with nasal diseases (i.e., AR, rhinosinusitis, rhinopolyp) in this study, including poor treatment adherence, lower education level, age ≥45 years and comorbid allergic rhinitis. The multivariate logistic regression analysis confirmed that risk of uncontrolled asthma in the group with treatment non-adherence for patients concomitant with nasal disease(s) was 5.2 times of that for patients with complete adherence. Similar finding was also obtained from asthma patients with AR that non-adherence had 3.6 folds more likely to have asthma uncontrolled, compared to treatment complete adherence. Previous studies demonstrated that insufficient treatment was prevalent among asthma patients due to overestimation of asthma control by themselves [27–31]. Only 17.1% of the asthmatic patients with comorbid nasal diseases in our study had completed asthma treatment adherence, even more, approximately 11.7% of them had not taken any asthma treatments, indicating that more efforts should be made to improve the health literacy of the importance of good treatment adherence and asthma control. Results that poor education level was also related to uncontrolled asthma was consistent with the findings in other studies that higher socioeconomic status or health literacy have been associated with good asthma control [32–34]. All above implies that patient educational intervention should be included in asthma management to increase treatment adherence and improve asthma control as well.

We should notice that AR played a more important role in the high proportion of uncontrolled asthma than any other nasal disease in this study. This was consistent with a publicized demonstration that AR adversely affected asthma control [29]. Whereas no association was found between other nasal diseases (e.g., rhinopolyp, rhinosinusitis) and asthma control in our study. Importantly, two risk factors of uncontrolled asthma identified for patients combined with AR were noteworthy: moderate-severe AR and perennial AR. Risk of uncontrolled asthma for patients with moderate-severe AR was found 2.2 times to that of patients with mild AR, suggesting that asthma will be harder to control among more severe AR patients.

Patients with perennial AR showed 1.5 folds more likely to have worse asthma control performance than patients with seasonal AR. According to ARIA (2008) [2], patients with perennial rhinitis have a greater bronchial reactivity than those with seasonal rhinitis. Considering asthma itself is chronic airway inflammation with airway hyper responsiveness [11,19] and AR and asthma coexist [2], from pathophysiology perspective, it is reasonable to explain that asthma is harder to control among asthma patients with perennial AR than seasonal AR. Chronic nasal obstruction and mouth breathing are common symptom of perennial AR [35]. In a 4-week trial of patients with perennial rhinitis and asthma, chronic nasal obstruction improved and mouth breathing decreased with active therapies. With related nasal function changes, daily asthma symptom was also reduced [35]. Results from our study was consistent with above studies’ common findings that perennial AR symptoms, chronic nasal obstruction and mouth breathing impact asthma. All above implies that nasal functions enhancement, especially for more severe AR and perennial AR, should be included in asthma management to improve asthma control.

One limitation of this study is that there is no definite conclusion between risk factors and uncontrolled asthma can be drawn as it is cross-sectional study. On the other hand, the recruitment of participants depended on patients’ clinical visits and their willingness to be surveyed which might result in selection study sizes. Patients willing to participate in the study might be more likely to adhere to physician’s advice or prescription, which may cause better adherence than those not in the study. Additionally, the judgment of treatment adherence was based on prescription refill instead of actual intake by patients, which may lead to adherence overestimated. It is also possible that the symptoms of asthma among patients enrolling from tertiary general hospitals were more severe than in ordinary asthma patients visiting lower grade hospitals. This may be a reason for the high proportion of uncontrolled asthma in this study. Therefore, interpreting the results needs caution that the uncontrolled rate might be overestimated for overall asthmatic patients. Participants were enrolled from November to June of the next year when nasal diseases (e.g., AR, rhinosinusitis) may be seasonal prevalent and the impact of AR on asthma control could be modified by the seasons such as winter and spring [36]. Despite its inherent limitations, this study provided a comprehensive and direct profile of the most recent asthma control status in Chinese adult patients combined with nasal diseases. The results in this specific population should be served as valuable references to guide further management of asthma control in patients with asthma and nasal diseases.

## Conclusions

The high prevalence of uncontrolled asthma among 1756 adult Chinese asthma patients comorbid with nasal disease was surveyed in this study, indicating that asthma management in such population is still a challenge. Poor treatment adherence, lower education level, age ≥45 years are risk factors contributing uncontrolled asthma and comorbid allergic rhinitis plays a more important role in the high proportion of uncontrolled asthma than other nasal diseases in this study. We believe that educating patients about asthma control, raising the awareness of asthma management and providing sufficient treatment will greatly contribute to improved quality of asthma management. The results also demonstrated that, in asthmatic patients with comorbid AR, it is possible to minimize the influence of AR on asthma control by improving nasal function, especially for more severe AR and perennial AR.

## Supporting information

S1 Checklist STROBE checklist. (DOCX)
