# Supplementary material for: The characteristic of asthma control among nasal diseases population: Results from a cross-sectional study
Source: PLoS One. 2018 Feb 22;13(2):e0191543. doi: 10.1371/journal.pone.0191543 (PMC5823402; doi:10.1371/journal.pone.0191543)
Supplement: S1 Checklist — (DOCX) [file pone.0191543.s001.docx]

STROBE Statement—checklist of items that should be included in reports of observational studies

|  | Item No. | Recommendation | Page  No. | Relevant text from manuscript |
| --- | --- | --- | --- | --- |
| **Title and abstract** | 1 | (*a*) Indicate the study’s design with a commonly used term in the title or the abstract | 2 | This is a cross-sectional study. |
|  |  | (*b*) Provide in the abstract an informative and balanced summary of what was done and what was found | 2 | 1756 asthma patients concomitant with nasal diseases aged ≥17 years and representing all regions of mainland China were surveyed. 56.4% of asthmatic patients with combined allergic rhinitis or rhinosinusitis or rhinopolyp remained uncontrolled. Concomitant without allergic rhinitis, younger age, better treatment adherence and higher education level might positively impact asthma control among asthmatic patients with combined nasal diseases. Perennial allergic rhinitis (OR=1.5, P=0.021), moderate-severe allergic rhinitis (OR=2.2, P=0.001) were all found to significantly increase the risk of uncontrolled asthma among asthma patients with combined allergic rhinitis. |
| Introduction | | | |  |
| Background/rationale | 2 | Explain the scientific background and rationale for the investigation being reported | 3-4 | Asthma and nasal diseases (consisting of rhinitis, rhinosinusitis, and rhinopolyp), are highly prevalent chronic respiratory diseases. Epidemiologic studies have consistently reported that asthma and nasal diseases often coexist. However, comprehensive evaluation of risks associated with poor asthma control in Chinese asthmatic patients concomitant with nasal diseases was still very limited. |
| Objectives | 3 | State specific objectives, including any prespecified hypotheses | 4 | This study aims to provide a comprehensive estimate of asthma control in Chinese asthma patients with combined nasal diseases, to explore the effect of kinds of nasal diseases on the asthma control, and to identify risk factors associated with uncontrolled asthmatic patients with combined nasal diseases. |
| Methods | | | |  |
| Study design | 4 | Present key elements of study design early in the paper | 5 | This nationwide cross-sectional observational study was conducted in China from November, 2012 to June, 2013. |
| Setting | 5 | Describe the setting, locations, and relevant dates, including periods of recruitment, exposure, follow-up, and data collection | 5-6 | This nationwide cross-sectional observational study was conducted in China from November, 2012 to June, 2013. The asthma patients aged ≥17 were consecutively enrolled from 48 tertiary general hospitals in 34 cities of different provinces across China, covering all territories and regions of mainland China except Tibet. Socio-demographic and clinical information, including family history of allergic disease, concomitant disease, asthma symptoms, asthma duration since diagnosis, asthma related treatments and tests, and asthma treatment adherence, was collected from participants’ medical charts during their visits. |
| Participants | 6 | (*a*) *Cohort study*—Give the eligibility criteria, and the sources and methods of selection of participants. Describe methods of follow-up  *Case-control study*—Give the eligibility criteria, and the sources and methods of case ascertainment and control selection. Give the rationale for the choice of cases and controls  *Cross-sectional study*—Give the eligibility criteria, and the sources and methods of selection of participants | 5-6 | This is a Cross-sectional study.  Eligibility criteria: 1. Had confirmed asthma for 3 months at least and had symptoms or took asthma medication during the past 12 months. 2. Aged ≥17 with concomitant AR or rhinosinusitis or rhinopolyp. 3. Patients who had intermittent asthma, COPD, bronchiectasis, bronchitis, cystic fibrosis, lung cancer or pneumonia or who were not able to fill in the questionnaires were excluded.  Sources: The asthma patients were consecutively enrolled from 48 tertiary general hospitals in 34 cities of different provinces across China, covering all territories and regions of mainland China except Tibet.  Methods of selection of participants: Asthma patients aged ≥2 years who visited outpatient clinics of respiratory disease for treatment, prescription refill and consultation were enrolled during this study period. |
|  |  | (*b*) *Cohort study*—For matched studies, give matching criteria and number of exposed and unexposed  *Case-control study*—For matched studies, give matching criteria and the number of controls per case |  |  |
| Variables | 7 | Clearly define all outcomes, exposures, predictors, potential confounders, and effect modifiers. Give diagnostic criteria, if applicable | 6-7 | Socio-demographic and clinical information, including family history of allergic disease, concomitant disease (e.g., rhinitis, allergic rhinitis), asthma symptoms, asthma duration since diagnosis, asthma related treatments and tests, and asthma treatment adherence, was collected from participants’ medical charts during their visits. Body Mass Index (BMI) was measured and classified as normal (18.5 ≤ BMI<24), lean (BMI<18.5), overweight (24≤BMI<28), and obesity (BMI≥28) based on Criteria Of Weight For Adults issued by National Health and Family Planning Commission of PRC in 2013. The five-item Asthma Control Test (ACT) questionnaire was used to assess the level of asthma control [23-24]. Asthma control was classified into controlled (ACT≥20) and uncontrolled (ACT≤19) [25]. The questionnaires was completed by participants during the interview and reviewed by physicians for completeness. |
| Data sources/ measurement | 8* | For each variable of interest, give sources of data and details of methods of assessment (measurement). Describe comparability of assessment methods if there is more than one group | 6 | Asthma treatment adherence was assessed by physicians through review of prescription refill in the past 3 months and was classified into 4 levels: complete adherence (≥90%), good adherence (70-89%), poor adherence (50-69%) and nonadherence (<50%). All participants had confirmed asthma for 3 months at least and had symptoms or took asthma medication during the past 12 months. Asthma diagnosis was confirmed by medical chart review at the enrolment visit. according to the Global Initiative for Asthma (GINA). The five-item Asthma Control Test (ACT) questionnaire was used to assess the level of asthma control. Asthma control was classified into controlled (ACT≥20) and uncontrolled (ACT≤19). The questionnaires were completed by participants during the interview and reviewed by physicians for completeness. |
| Bias | 9 | Describe any efforts to address potential sources of bias | 20 | No action to address potential bias but discussed it in the study limitation. |
| Study size | 10 | Explain how the study size was arrived at | 5 | Since the study was not testing any hypothesis, we calculated sample size based on the estimation of uncontrolled asthma rate. A sample size of 1500 was necessary (5% level of significance, two-sided) when we estimated uncontrolled asthma rate was 42% according to the result of Asia Pacific Asthma Insights and Management study. Data of 1756 asthma patients aged ≥17 with concomitant AR or rhinosinusitis or rhinopolyp among them will be used in this analysis. |

Continued on next page

| Quantitative variables | 11 | Explain how quantitative variables were handled in the analyses. If applicable, describe which groupings were chosen and why | 7, 10 | The quantitative variables such as age and BMI was categorized into several group. Age group (years): 17 – 29, 30 – 44, 45 – 59, 60 – 70, >70. BMI group (kg/m^2): 18.5≤ BMI<24 (normal), BMI<18.5 (lean), 24≤BMI<28 (overweight), BMI≥28 (obese). |
| --- | --- | --- | --- | --- |
| Statistical methods | 12 | (*a*) Describe all statistical methods, including those used to control for confounding | 7-8 | Descriptive statistical analysis of uncontrolled asthmatic patients’ number, rate and 95% CI were presented by demographic characteristics of different subgroups with nasal disease. To evaluate the association between clinical or disease categories, statistical analyses were performed using Chi-Square test to compare quantitative variables between 2 categories for uncontrolled asthma. Cochran-Armitage test was used to detect the trend of proportions of uncontrolled asthma in patients among more than 2 ordinal categories. A two times multivariate logistic regression model was used to identify the risk factors associated with uncontrolled asthma and to derive odds ratio (OR) and the 95% confidence interval of each factor. All related demographic characteristics and disease characteristics factors entered the multivariate logistic regression models. A two-tailed P-value of less than 0.05 was considered to be statistically significant. |
|  |  | (*b*) Describe any methods used to examine subgroups and interactions | 8 | statistical analyses were performed using Chi-Square test to compare qualitative variables between 2 categories for uncontrolled asthma. Cochran-Armitage test was used to detect the trend of proportions of uncontrolled asthma in patients among more than 2 ordinal categories. A two times multivariate logistic regression model was used to identify the risk factors associated with uncontrolled asthma and to derive odds ratio (OR) and the 95% confidence interval of each factor.  No interaction analysis was done. |
|  |  | (*c*) Explain how missing data were addressed |  | As there was very less missing data, no imputation was done and missing data was not included in the analysis and manuscript. |
|  |  | (*d*) *Cohort study*—If applicable, explain how loss to follow-up was addressed  *Case-control study*—If applicable, explain how matching of cases and controls was addressed  *Cross-sectional study*—If applicable, describe analytical methods taking account of sampling strategy |  | No methods on sampling strategy |
|  |  | (*e*) Describe any sensitivity analyses |  | No sensitivity analysis |
| Results | | | | |
| Participants | 13* | (a) Report numbers of individuals at each stage of study—eg numbers potentially eligible, examined for eligibility, confirmed eligible, included in the study, completing follow-up, and analysed |  | Since it’s one-time point cross-sectional study, there were no follow-up or more additional visit to report. |
|  |  | (b) Give reasons for non-participation at each stage |  | Since it’s one-time point cross-sectional study, there were no follow-up or more additional visit to report. |
|  |  | (c) Consider use of a flow diagram |  | Since it’s one-time point cross-sectional study, there were no follow-up or more additional visit to report. |
| Descriptive data | 14* | (a) Give characteristics of study participants (eg demographic, clinical, social) and information on exposures and potential confounders | 12, 13 | Patients aged ≥45 years had more proportions in uncontrolled asthma than younger age groups for asthmatic patients combined with AR (63.7% in age 45-59, 59.1% in age 60-70 and 67.5% in age >70 vs. 44.7% in age 17-29 and 54.41% in age 30-44) or rhinopolyp (65.0% in age 45-59 and 100.0% in age 60-70 vs. 47.1% in age 30-44), which indicated that age <45 years was significantly associated with better asthma control, relative to elder age groups. However, because of the sparse data of rhinopolyp group this result might be unreliable.  Compared to educational level of primary school and below, the group with education level of college and above had the lower proportion of uncontrolled asthma for asthmatic patients with AR (48.3% vs. 67.4%) or rhinosinusitis (29.6% vs. 70.0%). It showed that higher education level (i.e., college and above) was significantly conducive to control asthma better.  The lowest rates of uncontrolled asthma were observed in complete adherence groups for these three categories of patients respectively, compared to non-adherence group (AR: 41.5% vs. 79.6%; rhinosinusitis: 31.6% vs. 88.9%; with ≥2 nasal diseases: 47.8% vs. 81.0%).  Female patients were significantly worse in uncontrolled asthma, compared to male patients (81.0% vs. 36.0%). It was also found that smoking status had significant effect on uncontrolled rate of asthma in this subgroup. Uncontrolled rates were calculated as 71.9%, 37.5% and 0.0% for nonsmoker, ex-smoker and current smoker respectively, but this result might be irrational because of a paucity of data in smoker. |
|  |  | (b) Indicate number of participants with missing data for each variable of interest |  | As there was very less missing data, no imputation was done and missing data was not included in the analysis and manuscript. |
|  |  | (c) *Cohort study*—Summarise follow-up time (eg, average and total amount) |  | NA |
| Outcome data | 15* | *Cohort study*—Report numbers of outcome events or summary measures over time |  |  |
|  |  | *Case-control study—*Report numbers in each exposure category, or summary measures of exposure |  |  |
|  |  | *Cross-sectional study—*Report numbers of outcome events or summary measures | 8, 10-11 | According to ACT criteria, more than half (56.4%, 990/1756) of asthmatic patients with combined AR or rhinosinusitis or rhinopolyp remained uncontrolled (ACT≤19). uncontrolled asthma in groups with different characteristics is shown in Table 1. |
| Main results | 16 | (*a*) Give unadjusted estimates and, if applicable, confounder-adjusted estimates and their precision (eg, 95% confidence interval). Make clear which confounders were adjusted for and why they were included | 15, 17 | Table 3 and Table 5 presents the confounder-adjusted estimates for risk factors analysis. |
|  |  | (*b*) Report category boundaries when continuous variables were categorized | 7, 10 | Body Mass Index (BMI, kg/m2) was measured and classified as normal (18.5≤BMI<24), lean (BMI<18.5), overweight (24≤BMI<28), and obesity (BMI≥28) based on Criteria of Weight for Adults issued by National Health and Family Planning Commission of the People’s Republic of China in 2013. Table 1 also presents the category boundaries for age and BMI. |
|  |  | (*c*) If relevant, consider translating estimates of relative risk into absolute risk for a meaningful time period |  | NA |

Continued on next page

| Other analyses | 17 | Report other analyses done—eg analyses of subgroups and interactions, and sensitivity analyses |  | NA |
| --- | --- | --- | --- | --- |
| Discussion | | | | |
| Key results | 18 | Summarise key results with reference to study objectives | 3, 21 | 56.4% of asthmatic patients with combined allergic rhinitis or rhinosinusitis or rhinopolyp remained uncontrolled. Poor treatment adherence, lower education level, age ≥45 years are risk factors contributing uncontrolled asthma and comorbid allergic rhinitis plays a more important role in the high proportion of uncontrolled asthma than other nasal diseases. Perennial allergic rhinitis (OR=1.5, P=0.021), moderate-severe allergic rhinitis (OR=2.2, P=0.001) were all found to significantly increase the risk of uncontrolled asthma among asthma patients with combined allergic rhinitis. |
| Limitations | 19 | Discuss limitations of the study, taking into account sources of potential bias or imprecision. Discuss both direction and magnitude of any potential bias | 20 | One limitation of this study is that there is no definite conclusion between risk factors and uncontrolled asthma can be drawn as it is cross-sectional study. On the other hand, the recruitment of participants depended on patients’ clinical visits and their willingness to be surveyed which might result in selection study sizes. Patients willing to participate in the study might be more likely to adhere to physician’s advice or prescription, which may cause better adherence than those not in the study. Additionally, the judgment of treatment adherence was based on prescription refill instead of actual intake by patients, which may lead to adherence overestimated. It is also possible that the symptoms of asthma among patients enrolling from tertiary general hospitals were more severe than in ordinary asthma patients visiting lower grade hospitals. This may be a reason for the high proportion of uncontrolled asthma in this study. |
| Interpretation | 20 | Give a cautious overall interpretation of results considering objectives, limitations, multiplicity of analyses, results from similar studies, and other relevant evidence | 20-21 | The uncontrolled rate might be overestimated for overall asthmatic patients. Participants were enrolled from November to June of the next year when nasal diseases (e.g., AR, rhinosinusitis) may be seasonal prevalent and the impact of AR on asthma control could be modified by the seasons such as winter and spring [38]. Despite its inherent limitations, this study provided a comprehensive and direct profile of the most recent asthma control status in Chinese adult patients combined with nasal diseases. The results in this specific population should be served as valuable references to guide further management of asthma control in patients with asthma and nasal diseases. |
| Generalisability | 21 | Discuss the generalisability (external validity) of the study results | 20 | It is also possible that the symptoms of asthma among patients enrolling from tertiary general hospitals were more severe than in ordinary asthma patients visiting lower grade hospitals. This may be a reason for the high proportion of uncontrolled asthma in this study |
| Other information | |  | | |
| Funding | 22 | Give the source of funding and the role of the funders for the present study and, if applicable, for the original study on which the present article is based |  | Merck Sharp & Dohme (China) |

*Give information separately for cases and controls in case-control studies and, if applicable, for exposed and unexposed groups in cohort and cross-sectional studies.

**Note:** An Explanation and Elaboration article discusses each checklist item and gives methodological background and published examples of transparent reporting. The STROBE checklist is best used in conjunction with this article (freely available on the Web sites of PLoS Medicine at http://www.plosmedicine.org/, Annals of Internal Medicine at http://www.annals.org/, and Epidemiology at http://www.epidem.com/). Information on the STROBE Initiative is available at www.strobe-statement.org.
